# Cation leak: a common functional defect causing *HCN1* developmental and epileptic encephalopathy

**DOI:** 10.1093/braincomms/fcad156

**Published:** 2023-05-17

**Authors:** Chaseley E McKenzie, Ian C Forster, Ming S Soh, A Marie Phillips, Lauren E Bleakley, Sophie J Russ-Hall, Kenneth A Myers, Ingrid E Scheffer, Christopher A Reid

**Affiliations:** Florey Institute of Neuroscience and Mental Health, University of Melbourne, Parkville, VIC 3052, Australia; Florey Institute of Neuroscience and Mental Health, University of Melbourne, Parkville, VIC 3052, Australia; Florey Institute of Neuroscience and Mental Health, University of Melbourne, Parkville, VIC 3052, Australia; Florey Institute of Neuroscience and Mental Health, University of Melbourne, Parkville, VIC 3052, Australia; School of Biosciences, University of Melbourne, Parkville, VIC 3052, Australia; Florey Institute of Neuroscience and Mental Health, University of Melbourne, Parkville, VIC 3052, Australia; Department of Medicine, Epilepsy Research Centre, University of Melbourne, Austin Health, Heidelberg, VIC 3084, Australia; Department of Pediatrics, Faculty of Medicine, McGill University, Montreal, Montreal, Quebec H4A 3J1, Canada; Florey Institute of Neuroscience and Mental Health, University of Melbourne, Parkville, VIC 3052, Australia; Department of Medicine, Epilepsy Research Centre, University of Melbourne, Austin Health, Heidelberg, VIC 3084, Australia; Department of Paediatrics, University of Melbourne, Royal Children’s Hospital, Parkville, VIC 3052, Australia; Florey Institute of Neuroscience and Mental Health, University of Melbourne, Parkville, VIC 3052, Australia; Department of Medicine, Epilepsy Research Centre, University of Melbourne, Austin Health, Heidelberg, VIC 3084, Australia

**Keywords:** *HCN1*, developmental and epileptic encephalopathy, epilepsy, cation leak, electrophysiology

## Abstract

Pathogenic variants in *HCN1* are an established cause of developmental and epileptic encephalopathy (DEE). To date, the stratification of patients with *HCN1*-DEE based on the biophysical consequence on channel function of a given variant has not been possible. Here, we analysed data from eleven patients carrying seven different *de novo HCN1* pathogenic variants located in the transmembrane domains of the protein. All patients were diagnosed with severe disease including epilepsy and intellectual disability. The functional properties of the seven *HCN1* pathogenic variants were assessed using two-electrode voltage-clamp recordings in *Xenopus* oocytes. All seven variants showed a significantly larger instantaneous current consistent with cation leak. The impact of each variant on other biophysical properties was variable, including changes in the half activation voltage and activation and deactivation kinetics. These data suggest that cation leak is an important pathogenic mechanism in *HCN1*-DEE. Furthermore, published mouse model and clinical case reports suggest that seizures are exacerbated by sodium channel blockers in patients with *HCN1* variants that cause cation leak. Stratification of patients based on their ‘cation leak’ biophysical phenotype may therefore provide key information to guide clinical management of individuals with *HCN1*-DEE.

## Teaser

McKenzie *et al.* describe the functional analysis of seven *HCN1* pathogenic variants occurring in the transmembrane domain. Using two-electrode voltage-clamp analysis they have identified a common cation leak characteristic in all seven variants, suggesting that cation leak is an important pathogenic mechanism in *HCN1* developmental and epileptic encephalopathy.

## Graphical Abstract

**Graphical abstract:**
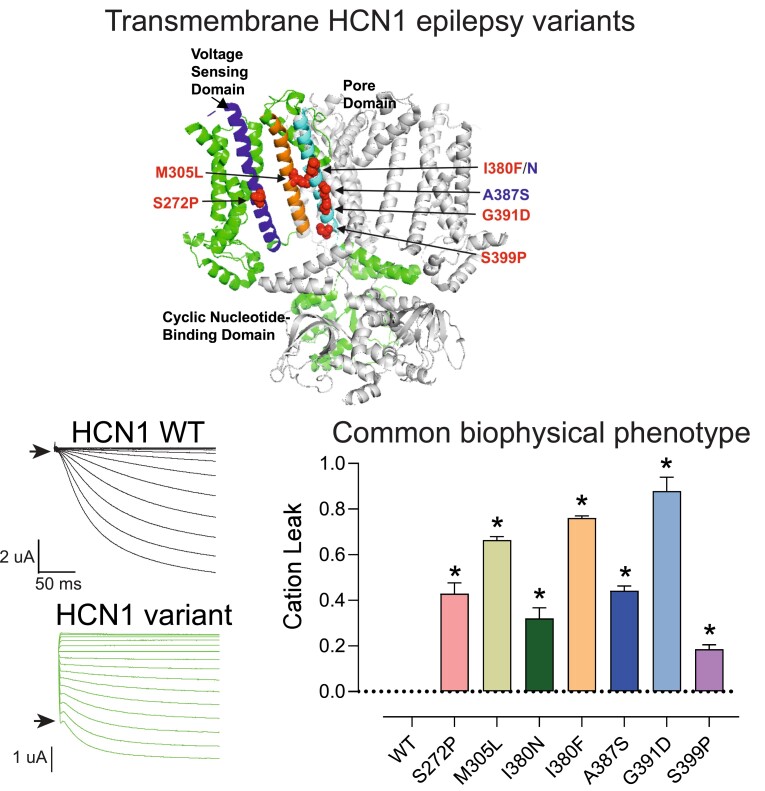

## Introduction

The developmental and epileptic encephalopathies (DEEs) are the most severe subgroup of epilepsies. They are characterised by drug-resistant seizures and epileptiform activity that result in developmental slowing or regression.^1^ Pathogenic variation in over eight hundred genes are now established causes of DEEs.^2^ Natural history studies are critical to establish the phenotypic spectrum and evolution of a given genetic DEE, including severity and range of epilepsy phenotypes, time course and pharmaco-responsiveness, and associated multimorbidities.^3^ Understanding the spectrum of natural histories of a genetic DEE can be further interpreted by considering the functional consequence of a pathogenic variant. This is perhaps best exemplified by DEEs caused by pathogenic variants in the sodium channel alpha-2 subunit gene, *SCN2A,* where stratification into variants causing either gain-of-function (GOF) or loss-of-function (LOF) has some ability to predict the natural history course including pharmacosensitivity.^4–7^ The categorisation of *SCN2A* DEE patients also carries critical implications for precision medicine approaches. Patient assignment based on the biophysical impact has also been established for other ion channel genes, including those encoding other sodium channels and GABA_A_ receptors.^8,9^ There is thus a crucial need to understand the functional consequence of a pathogenic variant in the development of precision medicines.

Pathogenic variants in *HCN1* are an established cause of DEE.^10–12^ Variants occurring in the transmembrane domains of HCN1 channels are more likely to cause severe disease.^10^ However, no clear correlation between the impact of a variant on the biophysical properties of the HCN1 protein and disease outcome has been established.^13^ This may be partially due to the incorrect assignment of GOF or LOF to a given variant. For example, the original classification of the recurrent *HCN1* M305L variant was LOF.^10^ We have since established cation leak as the pathophysiological basis of *HCN1*-DEE disease for the *HCN1* M305L variant.^14,15^ Our functional analysis in *Xenopus* oocytes revealed an uncoupling between the pore and voltage sensor domains, rendering the channel in a mostly open state.^14,15^ The cation leak phenotype was confirmed in mouse L5 pyramidal neurons carrying the homolog of the *HCN1* M305L variant.^14^ Despite this, the *HCN1* M305L variant has continued to be considered LOF by other research groups.^11^ Two technical issues could explain this. Firstly, ‘leaky’ cells are historically discarded in electrophysiological experiments as they are deemed to be of poor quality. Secondly, leak subtraction is frequently applied to electrophysiology data to remove background ‘leak’ predominantly through potassium channels. This has important ramifications for measuring changes in channel function in which constitutively open channels contribute significantly to the biophysical impact of a pathogenic variant. Here, we report functional analysis of two unstudied *HCN1* variants, together with re-analysis of five variants with some published functional data.^10–12,16–18^ All pathogenic variants were in the transmembrane domains of the HCN1 protein. A cation leak was established for all, suggesting that there is a common pathogenic mechanism underlying a subset of *HCN1* epilepsy.

## Materials and methods

### Selection of variants

We studied the biophysical properties of seven different *de novo HCN1* pathogenic variants located in the transmembrane domains of the HCN1 protein that are carried by eleven patients.

### Patient consent

The parents of new patient (Table 1, patient 8) and patient for which we supply additional clinical data (Table 1, patient 4) provided written informed consent for research participation. The research was approved by the Human Research Ethics Committee of Austin Health (H2007/02961) and the McGill University Health Centre Research Ethics Board (2018–3937). All other clinical data was summarised from published works as referenced in Table 1.

**Table 1:** Clinical summary of patients with pathogenic *HCN1* variants in transmembrane domains

Patient	Variant	Location	Inheritance	Sex	Epilepsy	Age at seizure onset	Initial seizure type	Triggers	Additional seizure types	Seizure Frequency	Development, other features	Drug resistance	Reference
1	S272P	S4	*de novo*	F, 16y^a^	DEE	8m	FS, HC	Fever	Clonic, atypical absence, focal	Several per month	Severe ID	Y	Nava *et al.*^12^ family 3
2	M305L	S5	*de novo*	F, 5y	EIDEE	2m	TCS	Fever	TCS, tonic, clonic, focal	Monthly	ID	Y	Marini *et al.*^10^ patient 10
3	M305L	S5	*de novo*	F, 14y^a^	EIDEE	3m	TCS	Fever	TCS, tonic, focal clonic	Monthly	Severe ID, microcephaly	Y	Marini *et al.*^10^ patient 11
4	I380N	S6	*de novo*	F, 5y	DEE	6m	Complex FS	Fever, illness, hot weather, stress, fatigue	Tonic clonic, FIAS, FBTC, tonic, SE	Monthly	Moderate to severe ID, autistic features, behavioural problems	Y	Scheffer *et al.*^16^ patient 9
5	I380F	S6	*de novo*	F, 7m^a^	EIDEE^b^	50d	FS, myoclonic seizures	Fever	Spasms, myoclonic, SE	40–50/day	GDD	Y	Wang *et al.*^18^ patient 231
6	I380F	S6	*de novo*	F, died 1y of uncontrolled seizures	EIDEE	2d	Tonic	Fever	Tonic, focal, SE	20–30/day	GDD, ventricular septal defect	Y	Xie *et al.*^11^ patient 2
7	A387S	S6	*de novo*	F, 24y^a^	DEE	0-12m	N/A	N/A	N/A	N/A	Severe ID, microcephaly, stereotypies > 10 years	Y	Lucariello *et al.*^17^ proband 4
8	A387S	S6	N/A	F, 7y	DEE	4m	FTBCS	N/A	FIAS with motor features (4 mths).	N/A	Profound ID, non-verbal, non-ambulatory	N	This study
9	G391D	S6	*de novo*	M, died 14m	EIDEE	30h	Tonic asymmetric, prolonged apnea & cyanosis; almost continuous at times	N/A	Asymmetric tonic ± clonic, prolonged apnoea, cyanosis	Daily	Severe ID; acquired microcephaly	Y	Marini *et al.*^10^ patient 12
10	G391D	S6	*de novo*	M, died 15m	EIDEE	48h	Grimace, R eye & head deviation, tonic asymmetric	N/A	Asymmetric tonic, cyanosis	Daily	Severe ID	Y	Marini *et al.*^10^ patient 13
11	S399P	C	*de novo*	M, 7y^a^	DEE	4m	Complex FS	Fever	TCS with apnoea, febrile	Daily	Severe ID	Y	Marini *et al.*^10^ patient 18

DEE = developmental and epileptic encephalopathy, EIDEE = early infantile developmental and epileptic encephalopathy, FS = febrile seizure, F = female, FIAS = focal impaired awareness seizure, FTBCS = focal to bilateral tonic-clonic seizure, GDD = global developmental delay, HC = hemiclonic seizure, ID = intellectual disability, IS = infantile spasms, M = male, m = months, N/A = not available, SE = status epilepticus, T = tonic seizure, TCS = tonic-clonic seizure, y = years.

a Age at time of original study (referenced).

b Although Wang *et al.* state that this patient had Dravet syndrome, this is not correct as Dravet syndrome does not have epileptic spasms and it would be exceptional for onset to be as young as 50 days, so we have termed this presentation as EIDEE.

### Site-directed mutagenesis and *in vitro* cRNA preparation

cDNA encoding a full-length transcript of wild-type human *HCN1* (RefSeq NM_021072.4 Ensemble database) was subcloned into the pGEMHE-MCS vector. Site-directed mutagenesis to create human *HCN1* variants was completed by GenScript Biotech (Piscataway, NJ, USA). All clones were verified by Sanger sequencing then linearized with *Nhe*I-HF (New England Biolabs) and purified using QIAquick PCR Purification Kit (QIAGEN). *In vitro* synthesis of cRNA was performed using linearized cDNA template and the mMessage mMachine® T7 transcription kit (Ambion, Thermo Fisher Scientific, Waltham, MA) and purified using RNeasy Mini Kit (QIAGEN). RNA integrity was assessed using NanoDrop Spectrophotometer and gel electrophoresis. cRNAs were stored at -80°C.

### *Xenopus* oocyte electrophysiology

#### Oocyte extraction

Adult female *Xenopus laevis* frogs were anaesthetized with 1.3 mg/ml tricaine methanosulfonate and oocytes were surgically removed via a small incision in the abdomen. Oocytes were defolliculated with 1.5 mg/ml collagenase for 2 h and rinsed with OR-2 solution (in mmol/L: 82.5 NaCl, 2 KCl, 1 MgCl_2_.6H_2_O, 5 HEPES, pH 7.4). Healthy mature oocytes stage V and VI were isolated for experiments.

#### Channel expression

cRNAs of 100 ng/µL coding for *HCN1* were manually injected into the oocytes to give a total injection volume of 50 nL. Injected oocytes were maintained in ND96 storage solution (in mmol/L: 96 NaCl, 2 KCl, 1 MgCl_2_.6H_2_0, 1.8 CaCl_2_.2H_2_O, 5 HEPES, 50 mg/L gentamicin, pH 7.4) at 17°C for two to three days to allow translation and trafficking of channels before experimentation.

#### Two-electrode voltage-clamp electrophysiology

Standard two-electrode voltage-clamp hardware was used (TEC-05X or TEC10X, NPI, Tamm, Germany) with series resistance (*R*_s_) compensation applied to ensure clamp accuracy when oocytes exhibited high functional expression. Oocytes were impaled with microelectrodes containing 3 mol/L KCl and with an input resistance between 0.2 and 1.5 MΩ. During experiments oocytes were continually perfused with high K^+^ solution (in mmol/L: 100 KCl, 1.8 CaCl_2_, 1 MgCl_2_, 10 HEPES, pH 7.4). All recordings were performed at 18–20°C. Voltage clamp control and data acquisition were obtained using pCLAMP8.10 software (Molecular Devices, USA). Oocytes were clamped at -30 mV holding potential and current–voltage relationships were generated using a voltage step protocol with incremental 10 mV steps from -120 mV to +20 mV for 2.5 s. Data were sampled at 200 µs/point and low pass filtered at 500 Hz. CsCl was dissolved in high K^+^ solution at a concentration of 10 mM before being applied during experiments. Oocytes used for experiments were selected if stable and had expression of the relevant channel.

#### Electrophysiological data analysis

Data were analysed using Prism 8.4.2 (GraphPad, USA) and Clampfit 10.4 (Molecular Devices, USA). The total steady-state current was measured over an approximately 200 ms interval at the end of the test pulse. For tail current analysis, baseline correction was applied after the current had reached a steady-state and the instantaneous current was estimated at the start of the repoad settled. This current was measured and fit with a form of the Boltzmann equation:


$$I_{\mathrm{tail}}=I_{\mathrm{tail}}^{\mathrm{off}}+I_{\mathrm{tail}}^{\max}/(1+e^{(V-V_{0.5})ze/kT}),$$

where $I_{\mathrm{tail}}^{\max}$ is the maximum instantaneous tail current and $I_{\mathrm{tail}}^{\mathrm{off}}$ is the offset reported by the fit (typically =0 with baseline correction), $V_{0.5}$ is the mid-point voltage, *z* the apparent valence of the charge moved, and kT/e = 25.3 mV at 20°C.

To characterise the channel activation kinetics, we fit a single exponential function to the activating current commencing after the initial inflexion. A double exponential fit (see Hung *et al.* 2021) was found to report reliable fits only for the WT and some of the constructs at extreme hyperpolarizing potentials. To characterise the channel deactivation kinetics, a single exponential fit was found to adequately describe the WT and WT+ variant channels for the monotonic relaxation phase.

### Statistical analysis

Data were analysed with Clampfit10.4 (Molecular Devices, USA) and Prism 8.4.2 (GraphPad, USA). Standard one-way ANOVA with Dunnett’s post-hoc correction was used for statistical comparisons to wild-type values. Significance was set at *P* < 0.05. Data were collected from at least two different batches of oocytes. All data points are shown as mean ± SEM unless otherwise stated.

## Results

### Clinical phenotypes of patients with pathogenic *HCN1* variants in the transmembrane domains

**Figure 1:**
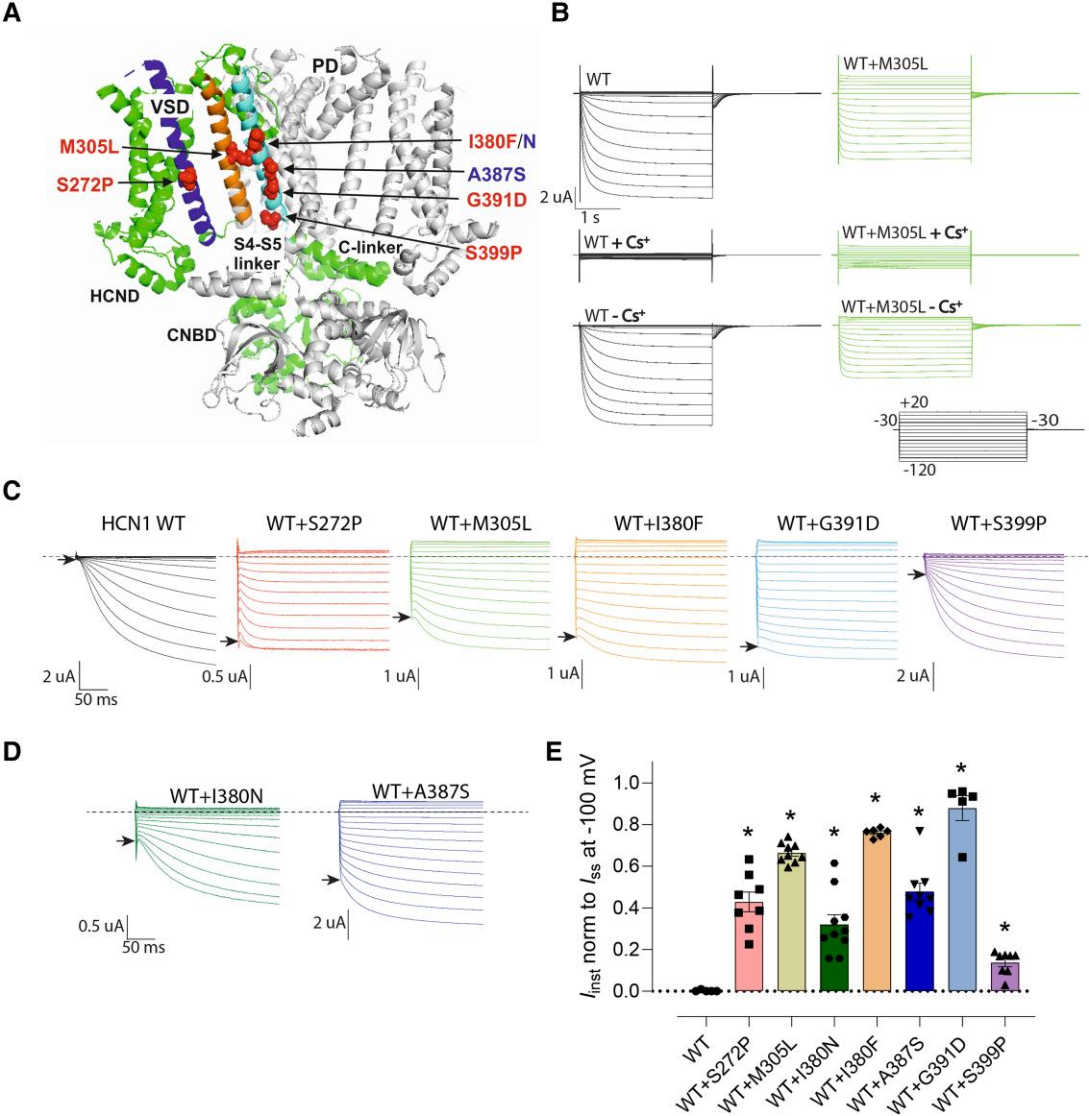
**Functional analysis of previously characterised *HCN1* pore domain variants revealed significant cation leak** (**A**) Location of variants analysed in this study (red spheres) within the structure of HCN1. Variants with previously published functional analysis shown in red text, and variants without prior functional analysis shown in blue text. The S4 helix of the voltage sensing domain (VSD) is shown is dark blue, and the S5 and S6 helices of the pore domain (PD) are shown in orange and light blue, respectively. The structure is based on the depolarised (closed) conformation of HCN1^26^ and was rendered using PyMOL (The PyMOL Molecular Graphics System, Version 2.3.4 Schrödinger, LLC). (**B**) Representative voltage clamp data from oocytes expressing HCN1 wild-type (WT) and co-expressed WT + M305L (top); in the presence of CsCl (middle) and with the CsCl traces subtracted from the corresponding traces at each test potential (bottom). Each dataset shows current traces in response to a series of 10 mV voltage steps (inset) from the holding potential (-30 mV) to test potentials in the range -120 mV to +20 mV. (**C**) An expanded view of HCN1 WT and co-expressed WT + S272P, WT + M305L, WT + I380F, WT + G391D, and WT + S399P traces with capacitive charging transients eliminated by subtracting records in Cs^+^ solution (see Materials and methods) and thus reveal the instantaneous and time-dependent activating components associated with the heterologously expressed channels. Arrows indicate instantaneous current component (*I*_inst_) for -120 mV step. Dotted line indicates 0 current reference. (**D**) Expanded view of WT + I380N and WT + A387S traces, with the same properties as those in (**C**). (**E**) Instantaneous current (normalised to steady-state current) at -100 mV for HCN1 WT and co-expressed WT + S272P, WT + M305L, WT + I380N, WT + I380F, WT + A387S, WT + G391D, and WT + S399P. *P* < 0.05 were considered significant and denoted *. Data were compared using one-way ANOVA with Dunnett’s post-hoc, compared to WT (See Supplementary Table 1 for detailed analysis and exact *P*-values).

We present the phenotypic data from eleven patients harboring seven different *de novo HCN1* pathogenic variants located in the transmembrane domains of the protein (Fig. 1A, Table 1). We report one new patient (patient 8) carrying the recurrent *HCN1* A387S variant and ten patients who have been previously published.^10–12,16–18^Table 1 summarizes the main clinical features of these patients. *HCN1*-DEE was severe in all individuals, with six patients having early infantile DEE (which now includes neonatal onset DEE) and five having DEE.^19^

Seizure onset occurred at a median age of 2.5 months (range 30 h to 8 months). At onset, seizures were triggered by fever in 7/11 patients. All patients developed additional seizure types, including focal, generalized and focal to bilateral tonic-clonic, myoclonic and absence seizures. EEG analysis typically showed multifocal epileptiform activity and diffuse slowing. All patients had intellectual disability, which was typically severe. Autistic features and behavioural problems were reported in some individuals. Two patients had microcephaly. Three patients died at ages between 12 and 15 months.

### Leak current is a common biophysical feature of all transmembrane *de novo* pathogenic *HCN1* variants tested

Two-electrode voltage-clamp recordings were made from *Xenopus laevis* oocytes to investigate the functional consequences of seven transmembrane-domain *HCN1* variants. These included five published variants (S272P, M305L, I380F, G391D, and S399P) and two published variants that have not been previously functionally characterized (A387S and I380N) (Fig. 1A). A 50:50 mix of each *HCN1* pathogenic variant RNA with WT HCN1 RNA was injected into oocytes to mimic their heterozygous status in patients. All five previously characterized variants displayed recordable currents (Fig. 1B, Supplementary Fig. 1). Cs^+^ is a known extracellular inorganic blocker of HCN channels and was used to distinguish between leak from endogenous channels and HCN channels.^20,21^ Cs^+^ (10 mM) effectively blocked WT and all WT+ variant HCN1-mediated currents (Fig. 1B, Supplementary Fig. 1). All subsequent analysis was completed on Cs^+^-subtracted currents.

An instantaneous jump in the voltage step-induced current is a measure of channels that are already open at the -30 mV holding potential. WT HCN1 channels generate a robust voltage-dependent current that can be measured at steady-state (*I*_ss_) with minimal instantaneous current (*I*_inst_), suggesting that the majority of channels are closed at a holding potential of -30 mV (Fig. 1C, arrow). In contrast, the S272P, M305L, I380F, G391D, and S399P *HCN1* pathogenic variants show marked *I*_inst_ reflective of mutant channels being open at the -30 mV holding potential (Fig. 1C, arrows). Similarly, co-expression of the newly functionally characterized A387S and I380N pathogenic variants with WT HCN1 also show marked *I*_inst_ (Fig. 1D, arrows). The average normalized *I*_inst_/*I*_ss_ highlights that *I*_inst_ is a significant proportion of the elicited current for all transmembrane *HCN1* pathogenic variants tested (Fig. 1E). This highlights that all transmembrane *HCN1* variants studied had a significant cation leak.

### Impact of transmembrane *de novo* pathogenic *HCN1* variants on other biophysical parameters

**Figure 2:**
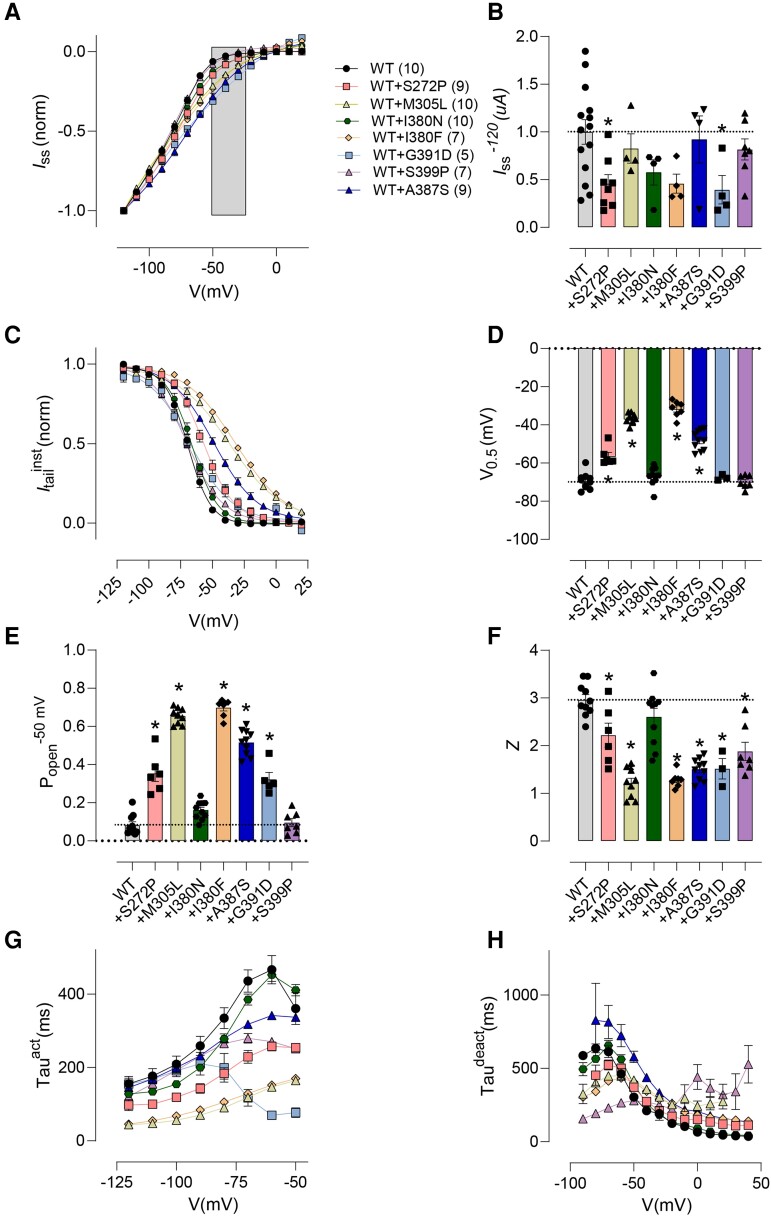
**Functional characterisation of two novel *HCN1* pore domain variants also revealed cation leak** (**A**) Pooled current–voltage (*I*–*V*) data normalised to -120 mV shows markedly weaker inward rectification for co-expressing oocytes [WT + S272P (*n* = 9), WT + M305L (*n* = 10), WT + I380N (*n* = 10), WT + I380F (*n* = 7), WT + A387S (*n* = 9), WT + G391D (*n* = 5), and WT + S399P (*n* = 7)] compared with WT (*n* = 10). Grey rectangle highlights a range of voltages at which a typical neuron. (**B**) Average of raw steady-state current (*I*_ss_) for WT + variant HCN1 channels measured at end of test pulse to -120 mV. (**C**) Normalised instantaneous tail currents for the co-injected variants indicated in (**B**). Data points were fit with a single Boltzmann function (see Materials and methods). (**D**) Half activation voltages (*V*_0.5_) for HCN1 WT and WT+ variant, respectively, reported by Boltzmann fits to data in (**C**). (**E**) Probability of channels being open at -50 mV for HCN1 WT compared to co-expressed WT+ variant obtained from normalised Boltzmann fits to data in (**C**). (**F**) Representation of the effective charge (*z*) reported by the Boltzmann fit for HCN1 WT and WT+ variant. (**G**) Mean activation time constant obtained by fitting the time-dependent component of activating current from wild-type and WT+ variant oocytes with a single-exponential function (see Materials and methods). (**H**) Deactivation time constant at potentials between -90 and +50 mV obtained by fitting the time-dependent component of deactivating current from wild-type and WT+ variant oocytes with a single-exponential function (see Materials and Methods). *P* < 0.05 were considered significant and denoted *. Data were compared using one-way ANOVA with Dunnett’s post-hoc, compared to WT (See Supplementary Tables 1–4 for detailed analyses and exact *P*-values).

Further analysis reveals that *HCN1* pathogenic variants impact on other biophysical parameters. The current–voltage relationships generated from normalised *I*_ss_ are presented in Fig. 2A. The shaded box around -50 mV highlights larger currents for all variants, consistent with the cation leak described above. The impact of *HCN1* pathogenic variant mediated *I*_ss_ at -120 mV normalised to within batch WT *I*_ss_ reveals that the majority of variants reduced expression levels in oocytes, although this was somewhat variable and may relate to variability inherent to the oocyte expression system (Fig. 2B). Current–voltage relationships generated from tail currents provide a measure of the channel open probability as a function of membrane potential (Fig. 2C). Fitting these data with a Boltzmann function (see Materials and Methods) revealed that the voltage of half activation (*V*_0.5_) was mainly right shifted (Fig. 2D). This resulted in an increase in the probability of opening at -50 mV (*P*_open_) for the majority of WT+ variant studied (Fig. 2E). The slope of the current–voltage relationship (*z*) was generally less steep (Fig. 2F). The impact of pathogenic *HCN1* variants on activation and deactivation was also variable with the time course mostly faster than HCN1 WT channels (Fig. 2G and H). In summary, all transmembrane domain pathogenic *HCN1* variants studied caused changes in one or more biophysical characteristics that increased the probability that the mutant channel would be open at membrane potentials where HCN1 WT channels would normally be closed, resulting in a cation leak.

## Discussion

Pathogenic variants in *HCN1* have emerged as an important cause of DEE.^10–12^ Marini *et al*. reported that *HCN1* variants associated with severe phenotypes tend to cluster within or close to the transmembrane domains. However, there has not been a clear functional correlate of the severe phenotype of *HCN1*-DEE.^13^ Here we report that seven *de novo HCN1* pathogenic variants which result in changes to amino acids within the transmembrane domains share a common mechanism of cation leak. Each of the eleven patients harbouring cation leak *HCN1* variants have DEEs with severe developmental impairment and drug-resistant seizures.^10–12,16–18^ We propose that *HCN1* pathogenic variants that result in cation leak are an important cause of *HCN1*-DEE.

Knock-in mouse models of *HCN1*-DEE based on human pathogenic variants have provided insight into the cellular and network basis of disease.^14,22^ The Hcn1 M294L mouse model (homolog of HCN1 M305L) recapitulates the disease phenotypes seen in patients, including spontaneous seizures and cognitive deficits.^14^ Whole-cell voltage clamp recordings from L5 pyramidal neurons reveal an HCN channel-mediated cation leak similar to that reported in the oocyte expression system.^14,15^ This leads to a more depolarised resting membrane potential in both L5 and CA1 pyramidal neurons, taking them closer to firing threshold.^14^ A similar depolarised mechanism is reported for CA1 pyramidal neurons in the Hcn1 G380D mouse model (homolog of HCN1 G391D).^22^

Importantly, both the Hcn1 M294L and Hcn1 G380D *HCN1*-DEE mouse models respond to standard anti-seizure medications (ASMs) in a similar manner.^14,22,23^ This includes seizure exacerbation induced by lamotrigine and phenytoin, both sodium channel blocking ASMs.^14,22^ In contrast, valproate reduced excitability on EEG (reduced spiking) in the Hcn1 M294L mouse and did not exacerbate seizures in the Hcn1 G380D mouse model.^14,22^ This mirrors reports from the parents of children with *HCN1*-DEE who carry the pathogenic variants modeled in the respective mice including seizure exacerbation with lamotrigine and phenytoin and some efficacy with valproate.^14,22^ Furthermore, sodium channel blocking ASMs also exacerbated seizures in the Hcn1 M142I mouse (homolog of *HCN1* M153I).^22^ Porro *et al*. reported a significant right shift in the voltage of half-activation of the M153I variant, increasing the probability of channels being open at depolarised potentials which would contribute to a cation leak.^13^ Collectively, these data suggest that sodium channel blocking ASMs should be avoided in *HCN1*-DEE where pathogenic variants cause cation leak. These data also have implications for precision medicine approaches with strategies aimed at reducing cation leak likely to be beneficial for patients with *HCN1*-DEE.

*HCN1* pathogenic variants are associated with a broad phenotypic spectrum including milder phenotypes such as idiopathic generalised epilepsies and genetic epilepsy with febrile seizures plus.^10^ Our current functional data focuses on *HCN1* pathogenic variants that cause cation leak associated with *HCN1*-DEE. Our current knowledge of *HCN1* variants that do not cause cation leak is limited. Future studies investigating biophysical properties of *HCN1* pathogenic variants associated with milder phenotypes are needed, included rodent and stem cell models, complemented by pharmacological studies.

Interestingly, there is phenotypic variability in patients with *HCN1*-DEE which correlates in part with the severity of the cation leak. The basis of these differences is likely to be complex. Factors could include subtle differences in other biophysical properties and variant-dependent differences in channel trafficking, as well as patients’ genetic background.

The biophysical mechanisms underlying cation leak caused by *HCN1* pathogenic variants are likely to be multifactorial. A large depolarising shift in the activation voltage as described above for the *HCN1* M153I variant will result in more channels being open at depolarised potentials and contribute to an inward cation current.^10,13^ Additionally, our work on the *HCN1* M305L variant suggests a decoupling of the voltage-sensor and pore domain resulting in a constitutively open pore.^14,15^ Recent biophysical models of voltage to pore coupling in *HCN1* suggest that the pore is essentially ‘spring-loaded’, and that the movement of the voltage sensor frees space for the channel to open.^24,25^ We, therefore, predict that the *HCN1* pathogenic variants destabilise a ‘scaffold’, which would normally keep the channel closed at depolarized potentials, resulting in the uncoupling of voltage to pore domains and leading to a cation leak. Consistent with this, in addition to M305L, the variants I380N/F, G391D, and A387S also show reduced inward rectification at depolarised potentials. Although we have focused on transmembrane *HCN1* variants, variants in other regions of the protein are also likely to cause changes that result in increased cation flow at more depolarised potentials. Additionally, future work is needed to see how *HCN1* variants impact heteromeric channels including those formed with HCN2 channels and whether these data can further refine the genotype-phenotype relationship in *HCN1*-DEE.

For the DEEs, understanding the impact of each patient’s pathogenic variant is critical. This not only includes the gene itself, but also how specific pathogenic variants impact protein function. The correct assignment of patients into functional groups has distinct ramifications for diagnosis and optimisation of therapy. This includes use of current ASMs and the development of precision medicines. Here, we identify cation leak as an important cause of *HCN1*-DEE. Precision therapies targeting cation leak may provide a common therapeutic mechanism for patients with this severe DEE.

## Supplementary Material

fcad156_Supplementary_Data

## Data Availability

Raw data are available upon request from the corresponding author.

## Abbreviations

ASM =: anti-seizure medication
DEE =: developmental and epileptic encephalopathy
EIDEE =: early infantile developmental and epileptic encephalopathy
GOF =: gain-of-function
HCN channel =: hyperpolarization-activated cyclic nucleotide-gated ion channel
LOF =: loss-of-function
WT =: wild-type
