# Supplementary material for: Cation leak: a common functional defect causing HCN1 developmental and epileptic encephalopathy
Source: Brain Commun. 2023 May 17;5(3):fcad156. doi: 10.1093/braincomms/fcad156 (PMC10231804; doi:10.1093/braincomms/fcad156)
Supplement: fcad156_Supplementary_Data [file fcad156_supplementary_data.pdf]

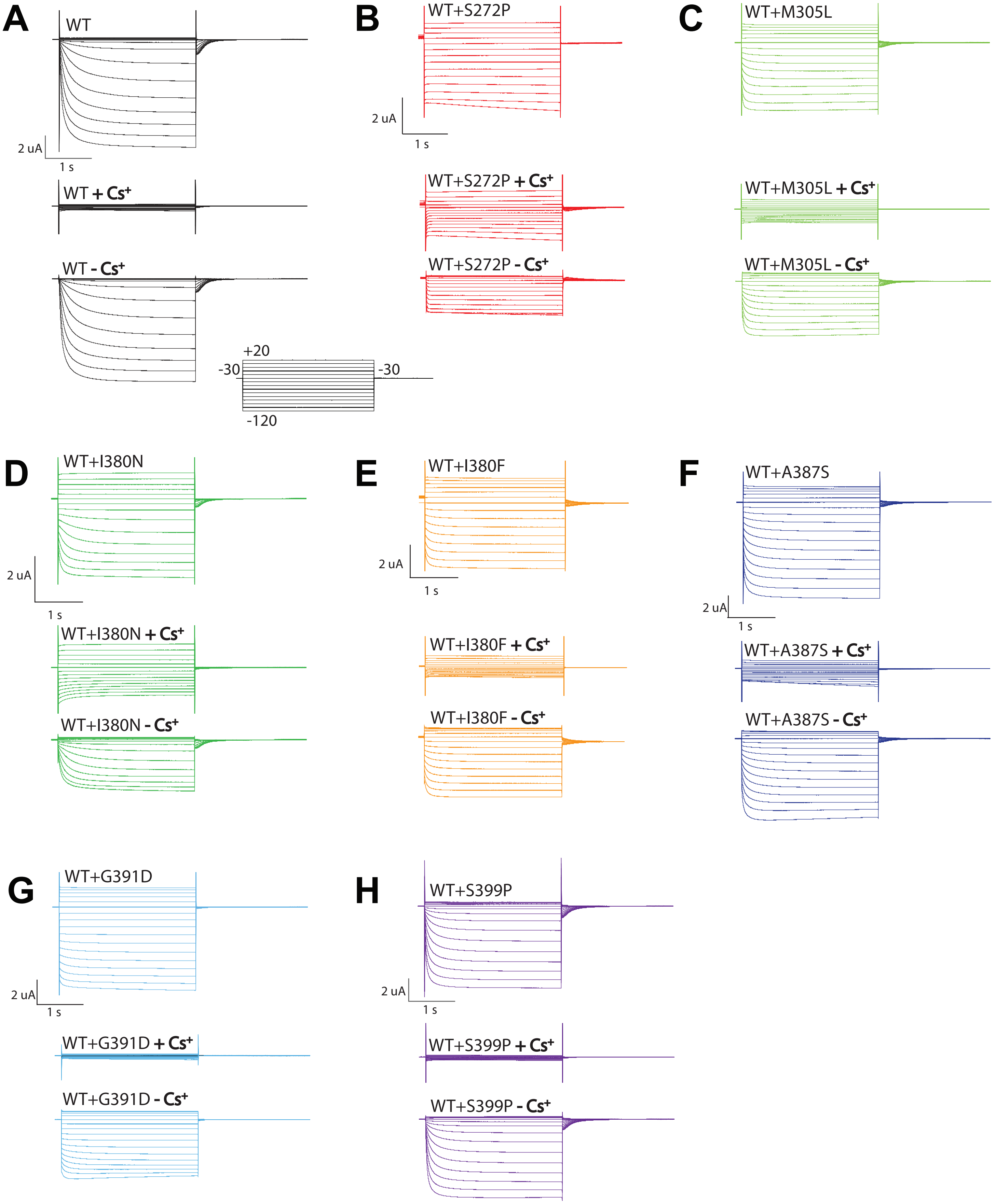

A
WT
2 uA
1 s
WT + Cs+
WT - Cs+
+20
-30
-30
-120
B
WT+S272P
2 uA
1 s
WT+S272P + Cs+
WT+S272P - Cs+
C
WT+M305L
WT+M305L + Cs+
WT+M305L - Cs+
D
WT+I380N
2 uA
1 s
WT+I380N + Cs+
WT+I380N - Cs+
E
WT+I380F
2 uA
1 s
WT+I380F + Cs+
WT+I380F - Cs+
F
WT+A387S
2 uA
1 s
WT+A387S + Cs+
WT+A387S - Cs+
G
WT+G391D
2 uA
1 s
WT+G391D + Cs+
WT+G391D - Cs+
H
WT+S399P
2 uA
1 s
WT+S399P + Cs+
WT+S399P - Cs+

Supplementary Figure 1. Representative functional data of pore domain *HCN1* variants. (**A**) Representative voltage clamp data from oocytes expressing HCN1 wild-type (WT), (**B**) co-expressed WT+S272P, (**C**) WT+M305L, (**D**) WT+I380N, (**E**) WT+I380F, (**F**) WT+A387S, (**G**) WT+G391D, (**H**) and co-expressed WT+S399P: in the presence of CsCl (middle) and with the CsCl traces subtracted from the corresponding traces at each test potential (bottom). Each dataset shows current traces in response to a series of 10 mV voltage steps (inset) from the holding potential (−30 mV) to test potentials in the range −120 mV to +20 mV.

Supplementary table 1: Summary of electrophysiology parameters (expressed as mean ± s.e.m) of hHCN1 wild-type and variants.

	**Wild-type**	**WT+S272P**	**WT+M305L**	**WT+I380N**	**WT+I380F**	**WT+A387S**	**WT+G391D**	**WT+S399P**
**Number of oocytes**	10	9	10	10	7	9	5	7
**$V_{0.5}$ (mV)**	-70.7 ± 1.58	-56.8 ± 0.63, ****P < 0.0001	-36.7 ± 0.59, ****P < 0.0001	-65.87 ± 0.62, P = 0.6805	-31.65 ± 0.49, ****P < 0.0001	-50.35 ± 0.59, ****P < 0.0001	-66.09 ± 1.49, P = 0.9837	-69.73 ± 1.31, P = 0.9998
**z valence**	2.70 ± 0.08	2.04 ± 0.09, **P = 0.0069	1.17 ± 0.03, ****P < 0.0001	2.48 ± 0.13, P = 0.2715	1.27 ± 0.03, ****P < 0.0001	1.51 ± 0.05, ****P < 0.0001	1.07 ± 0.06, ****P < 0.0001	1.89 ± 0.19, ****P < 0.0001
**I_{inst} (µA) normalized to I_{ss} at -100 mV**	0 ± 0	-0.43 ± 0.05, ****P < 0.0001	-0.66 ± 0.014, ****P < 0.0001	-0.32 ± 0.047, ****P < 0.0001	-0.76± 0.009, ****P < 0.0001	-0.44 ± 0.02, ****P < 0.0001	-0.88 ± 0.06, ****P < 0.0001	-0.19 ± 0.019, P = 0.1103
**Prob open at -50 mV**	0.1 ± 0.016	0.35 ± 0.042, ****P < 0.0001	0.66 ± 0.014, ****P < 0.0001	0.17 ± 0.017, P = 0.0373	0.7 ± 0.017, ****P < 0.0001	0.49 ± 0.026, ****P < 0.0001	0.32 ± 0.036, ****P < 0.0001	0.1 ± 0.02, P = 0.9994
**I^{-120} (µA)**	1.0 ± 0.13	0.46 ± 0.094, P = 0.0134	0.83 ± 0.15, P = 0.9633	0.58 ± 0.13, P = 0.2708	0.46 ± 0.1, P = 0.0828	0.92 ± 0.25, P = 0.9995	0.39 ± 0.15, P = 0.0395	0.82 ± 0.11, P = 0.8758

** P < 0.01, *** P < 0.001, **** P < 0.0001 from one-way ANOVA with Dunnett's post-hoc, compared to WT.

s.e.m = standard error of the mean
hHCN1 = human hyperpolarization-activated cylic nucleotide-gated channel 1
V0.5 = midpoint voltage
mV = millivolt
Z = apparent valence of the charge moved
I = current
Prob = probability

Supplementary table 2: Statistical comparison of tau activation (in milliseconds, expressed as mean ± s.e.m).

**Voltage**	**Wild-type**	**WT+S272P**	**WT+M305L**	**WT+I380N**	**WT+I380F**	**WT+A387S**	**WT+G391D**	**WT+S399P**
**-50 mV**	360.250 ± 43.522	254.071 ± 6.356, ****P* = 0.0006	164.605 ± 5.452, *****P* < 0.0001	410.426 ± 15.233, *P* = 0.1574	169.776 ± 6.059, *****P* < 0.0001	336.858 ± 7.328, *P* = 0.8770	76.732 ± 13.270, *****P* < 0.0001	250.920 ± 9.546, ****P* = 0.0008
**-60 mV**	466.540 ± 38.631	257.226 ± 12.162, *****P* < 0.0001	146.522 ± 6.035, *****P* < 0.0001	452.330 ± 17.799, *P* = 0.9867	151.676 ± 7.034, *****P* < 0.0001	341.801 ± 7.935, *****P* < 0.0001	69.652 ± 8.876, *****P* < 0.0001	270.362 ± 8.855, *****P* < 0.0001
**-70 mV**	435.466 ± 30.545	228.809 ± 17.262, *****P* < 0.0001	118.740 ± 6.205, *****P* < 0.0001	384.930 ± 14.809, *P* = 0.1558	128.659 ± 8.258, *****P* < 0.0001	317.370 ± 9.804, ****P* = 0.0001	117.117 ± 22.731, *****P* < 0.0001	279.678 ± 13.044, *****P* < 0.0001
**-80 mV**	334.474 ± 28.493	183.606 ± 15.488, *****P* < 0.0001	90.401 ± 6.763, *****P* < 0.0001	278.179 ± 9.992, *P* = 0.0707	105.811 ± 7.951, *****P* < 0.0001	278.857 ± 12.516, *P* = 0.1145	199.116 ± 38.881, *****P* < 0.0001	265.008 ± 6.814, *P* = 0.0430
**-90 mV**	258.835 ± 26.277	142.733 ± 12.919, *****P* < 0.0001	70.029 ± 6.573, *****P* < 0.0001	199.430 ± 6.939, *P* = 0.0140	84.116 ± 7.295, *****P* < 0.001	231.933 ± 11.592, *P* = 0.6140	210.092 ± 30.035, *P* = 0.1796	230.487 ± 8.286, *P* = 0.6157
**-100 mV**	208.507 ± 22.218	118.594 ± 10.882, *****P* < 0.0001	56.128 ± 6.173, *****P* < 0.0001	154.203 ± 5.059, ***P* = 0.0096	67.552 ± 6.439, *****P* < 0.0001	199.639 ± 15.670, *P* = 0.9941	190.937 ± 15.804, *P* = 0.9115	191.994 ± 11.268, *P* = 0.9024
**-110 mV**	176.551 ± 20.301	99.914 ± 11.371, ****P* = 0.002	47.289 ± 6.023, *****P* < 0.0001	133.986 ± 5.692, *P* = 0.0414	54.642 ± 5.545, *****P* < 0.0001	168.893 ± 13.455, *P* = 0.9958	168.013 ± 12.035, *P* = 0.9972	155.418 ± 11.986, *P* = 0.7076
**-120 mV**	155.488 ± 20.359	97.489 ± 14.951, *P* = 0.0184	44.268 ± 2.748, *****P* < 0.0001	127.617 ± 5.996, *P* = 0.4304	45.362 ± 4.956, ****P* = 0.0001	145.211 ± 16.418, *P* = 0.9916	155.020 ± 16.282, *P* > 0.9999	121.099 ± 10.561, *P* = 0.3510

** P < 0.01, *** P < 0.001, **** P < 0.0001 from one-way ANOVA with Dunnett's post-hoc, compared to WT.

Supplementary table 3: Statistical comparison of tau deactivation (in milliseconds, expressed as mean ± s.e.m).

Voltage	Wild-type	WT+S272P	WT+M305L	WT+I380N	WT+I380F	WT+A387S	WT+S399P
**20 mV**	45.681 ± 2.224	120.301 ± 20.096, P = 0.2493	273.951 ± 50.851, ****P < 0.0001	54.530 ± 4.065, P = 0.9977	154.369 ± 5.386, **P = 0.0032	159.306 ± 21.702, P = 0.0741	320.932 ± 70.831, ****P < 0.0001
**10 mV**	53.856 ± 2.800	134.089 ± 20.331, P = 0.2322	259.080 ± 42.938, ****P < 0.0001	68.805 ± 3.610, P = 0.9918	166.531 ± 5.987, **P = 0.038	177.912 ± 21.821, P = 0.0542	365.988 ± 59.268, ****P < 0.0001
**0 mV**	65.553 ± 3.977	152.543 ± 14.906, P = 0.4263	241.998 ± 35.177, **P = 0.0102	90.780 ± 8.138, P = 0.9834	180.760 ± 7.553, P = 0.0653	206.501 ± 19.269, P = 0.1966	441.335 ± 84.322, ****P < 0.0001
**-10 mV**	104.241 ± 17.596	149.820 ± 11.413, P = 0.7952	296.254 ± 91.340, ***P = 0.0007	114.329 ± 7.349, P = 0.9986	202.707 ± 10.125, P = 0.0663	218.218 ± 41.078, P = 0.1013	
**-20 mV**	124.570 ± 11.175	172.312 ± 13.647, P = 0.0426	253.142 ± 8.225, ****P < 0.0001	131.655 ± 5.267, P = 0.9744	260.278 ± 14.406, ****P < 0.0001	263.513 ± 15.498, ****P < 0.0001	221.005 ± 17.209, ****P < 0.0001
**-30 mV**	191.884 ± 46.236	211.214 ± 14.907, P = 0.9998	305.108 ± 3.965, P = 0.0788	181.775 ± 4.459, P = 0.9977	298.079 ± 13.905, P = 0.0750	333.821 ± 7.211, P = 0.0339	250.920 ± 9.546, P = 0.5127
**-40 mV**	210.819 ± 46.236	274.108 ± 15.028, **P = 0.0113	358.258 ± 8.520, ****P < 0.0001	261.034 ± 4.107, P = 0.0167	343.550 ± 19.390, ****P < 0.0001	437.951 ± 12.046, ****P < 0.0001	270.362 ± 8.855, **P = 0.0036
**-50 mV**	304.317 ± 18.117	371.228 ± 13.370, P = 0.0669	419.491 ± 17.341, **P = 0.0013	390.446 ± 13.910, **P = 0.0042	406.233 ± 24.581, ***P = 0.0007	578.284 ± 39.109, ****P < 0.0001	279.678 ± 13.044, P = 0.9343
**-60 mV**	461.131 ± 36.245	499.757 ± 23.750, P = 0.7824	447.484 ± 17.341, P = 0.9997	561.265 ± 31.689, P = 0.0529	434.434 ± 18.181, P = 0.9904	722.481 ± 76.082, ****P < 0.0001	265.008 ± 6.814, ****P < 0.0001
**-70 mV**	613.797 ± 40.584	523.016 ± 33.813, P = 0.6551	448.106 ± 16.437, P = 0.0321	656.878 ± 33.702, P = 0.8769	414.063 ± 18.679, ***P = 0.0011	815.124 ± 113.019, P = 0.0133	230.487 ± 8.286, ****P < 0.0001
**-80 mV**	635.991 ± 30.328	453.328 ± 68.519, P = 0.0830	402.039 ± 11.834, **P = 0.0027	563.395 ± 14.004, P = 0.6364	341.467 ± 14.858, ****P < 0.0001	827.191 ± 251.545, P = 0.0844	191.994 ± 11.268, ****P < 0.0001
**-90 mV**	587.274 ± 23.733		325.593 ± 65.642, ****P = 0.0002	493.847 ± 45.126, P = 0.2574	303.807 ± 28.610, ****P < 0.0001		155.418 ± 11.986, ****P < 0.0001

** P < 0.01, *** P < 0.001, **** P < 0.0001 from one-way ANOVA with Dunnett's post-hoc, compared to WT.

Supplementary table 4: Statistical comparison of I_{ss}(norm) for -30 to -50 mV (area shaded in grey in Fig. 2A).

Voltage	WT+S272P	WT+M305L	WT+I380N	WT+I380F	WT+A387S	G391D	WT+S399P
**-30 mV**	*P* = 0.1671	****$P < 0.0001$	*P* = 0.0881	****$P < 0.0001$	****$P < 0.0001$	****$P < 0.0001$	*P* = 0.8456
**-40 mV**	***P* = 0.0082	****$P < 0.0001$	*P* = 0.0315	****$P < 0.0001$	****$P < 0.0001$	****$P < 0.0001$	*P* = 0.9999
**-50 mV**	****P* = 0.0005	****$P < 0.0001$	*P* = 0.0149	****$P < 0.0001$	****$P < 0.0001$	****$P < 0.0001$	*P* = 0.9388

** $P < 0.01$, *** $P < 0.001$, **** $P < 0.0001$ from one-way ANOVA with Dunnett's post-hoc, compared to WT.
